# Fatality Chooses Its Path Through the Orbit: A Study of Rhino-Orbito-Cerebral Mucormycosis as a Complication of COVID-19 Infection

**DOI:** 10.7759/cureus.31822

**Published:** 2022-11-23

**Authors:** Samreen Mehfooz, Neelima Mehrotra, Tarim Usmani, Vivek Raj

**Affiliations:** 1 Department of Ophthalmology, Shri Ram Murti Smarak Institute of Medical Sciences, Bareilly, IND; 2 Department of Radiodiagnosis, Career Institute of Medical Sciences & Hospital, Lucknow, IND

**Keywords:** steroids in covid 19, covid-19 and diabetes, covid-19, otolaryngology, long covid, post covid-19 sequelae, covid-19-associated mucormycosis, comorbidities and covid-19, covid-19 in ophthalmology, post covid-19 rhino-orbito-cerebral mucormycosis (rocm)

## Abstract

Introduction and aim

Mucormycosis is a rare but serious angio-invasive infection caused by a group of fungi called mucormycetes and it mainly affects people who are immunocompromised, or patients already infected with other diseases. The dreaded mucormycosis infection has recently gained gross ill-repute for having claimed many lives in coronavirus disease (COVID-19) and/or post-COVID-19 patients. Hence a need was felt to study the development of mucormycosis in COVID-19 patients to better prevent and treat this fungal infection in anticipated future waves of the pandemic. This study also aims to establish an association between COVID-19 positivity, systemic comorbidities, and treatment modalities with the possibility of occurrence of vision and life-threatening mucor infection of the nose, paranasal sinuses, orbit, and brain.

Methods

This is a hospital-based, retrospective, case-control study. The study reviewed case files of all patients diagnosed with rhino-orbito-cerebral mucormycosis (ROCM) from April 1, 2021, to May 31, 2021. A set of age-matched COVID-19-positive patients hospitalized during the study period with moderate to severe disease were recruited as controls. We addressed factors that could be associated with the development of fungal infection and studied the period between COVID-19 positivity and the onset of ROCM.

Results

The age of patients in both groups ranged from 40-60 years with 13 females and 17 males. A statistically significant correlation (p-value = 0.032) was found between positive reverse transcription-polymerase chain reaction (RT-PCR) history and use of intravenous (IV) corticosteroids (11 [73.3%] cases and all controls). The mean duration from COVID-19 positivity to the presentation of mucormycosis was 12.10±7.27 days. Uncontrolled blood sugar was found to be the most significant correlation (p-value = 0.003). Mucormycosis is 13.678 times more likely in people with abnormal hemoglobin A1c (HbA1c). Co-morbidities like anemia, chronic kidney disease (CKD), coronary artery disease (CAD), and leukemia were found in controls, but none of these conditions were seen in patients who developed mucormycosis.

Conclusion

Judicious use of steroids and strict control of blood sugar levels should be emphasized in the management of COVID-19 patients.

## Introduction

The term mucormycosis stands for the acute or subacute rapidly progressing infections caused by the angio-invasive fungi of the order Mucorales [1,2]. Mucormycosis is a rare but serious angio-invasive infection caused by a group of fungi called mucormycetes and it mainly affects people who are immunocompromised or patients already infected with other diseases. The fungus is liable to cause devastating disease in patients with poorly controlled diabetes mellitus and other immunocompromised patients and often causes significant morbidity and mortality. The most common clinical manifestation is that of rhino-orbito-cerebral mucormycosis (ROCM) [3]. The dreaded mucormycosis infection has recently gained gross ill-repute for having claimed many lives in coronavirus disease (COVID-19) and/or post-COVID-19 patients.

Coronavirus infection caused by the novel severe acute respiratory syndrome coronavirus-2 (SARS-CoV-2) is associated with a wide variety of patterns, ranging from mild disease to life-threatening conditions. Patients with medical co-morbidities (e.g., diabetes mellitus, chronic obstructive pulmonary disease) and certain immunocompromised conditions (e.g., corticosteroid therapy, ventilation, intensive care unit stay) are at an elevated risk for developing opportunistic infections, Rhino-orbito-cerebral mucormycosis (ROCM) being one of them. The second wave of the pandemic has overwhelmingly presented with catastrophes including loss of eyes and lives.

Secondary infections are more commonly seen in hospitalized, severely ill COVID-19 patients, with an incidence ranging between 10 and 30%, fungal infections being 10 times more common among such.

The incidence of mucormycosis recorded globally varies from 0.005 to 1.7 per million population [4]. However, its prevalence in the Indian population is 0.14 per 1000, which is about 80 times higher than in developed countries [5]. The fatality rate of mucormycosis is 46% globally [6]. Factors like intracranial extension, orbital involvement, and irreversible immune suppression increase fatality to as high as 50% to 80% [7]. High suspicion of this disease must be considered in patients who are immunocompromised.

## Materials and methods

The study aimed to identify the risk factors involved in the development of mucormycosis. A hospital-based, retrospective, case-control study was conducted at Shri Ram Murti Smarak Institute of Medical Sciences, Bareilly. This study reviewed the case files of all consecutive patients who were diagnosed with ROCM from April 1, 2021, to May 31, 2021. A set of age-matched COVID-19-positive patients hospitalized with moderate to severe disease were taken as controls.

Informed consent was obtained from all the participants. The study adhered to the tenets of the Declaration of Helsinki and was approved by the appropriate institutional review boards.

Rhino-orbito-cerebral mucormycosis was diagnosed clinically based on various symptoms and signs such as nasal stuffiness, epistaxis, nasal discharge, facial pain, facial paresthesia, proptosis, sudden ptosis, periocular edema, restriction of extraocular muscles (EOM), conjunctival chemosis, diminution of vision. The diagnosis was confirmed by radiological investigation, i.e. contrast-enhanced MRI of paranasal sinuses, orbit, and brain, and direct microscopy, culture, and histopathology of the endoscopy-guided deep nasal swab. Laboratory tests included a complete hemogram, blood sugar, and renal function tests. The disease was further staged according to the proposed staging of ROCM [8].

Information regarding the presenting complaints, visual acuity, and detailed ocular examination of patients with mucormycosis was noted. All the patients had a history of COVID-19 positivity, though many at presentation with ROCM were negative for COVID-19 infection by reverse transcription-polymerase chain reaction (RT-PCR) testing.

All the patients presenting with ROCM were under consultation from an otorhinolaryngologist, pulmonologist, neurologist, and ophthalmologist. Nasal endoscopy-guided exploration and debridement were primarily used for the management of these patients. Additional systemic intravenous antifungals in the form of liposomal amphotericin B (5 mg/kg body weight) or posaconazole (loading dose 300 mg twice a day on the first day, maintenance dose 300 mg once a day starting on the second day) were added for all the patients. Oral posaconazole (300 mg) was added at the time of resolution. All medications were started and tapered depending on clinical features and response to treatment. The patients were subjected to serial imaging studies or nasal endoscopy to know their disease status. Repeat sinus and/or local tissue debridement was done in patients with worsening disease. The resolution was taken to be evident in clinical improvement and radiological investigation. The resolution was confirmed by a negative sinus biopsy and normal parameters.

Since most of these patients had a history of diabetes mellitus, the values of hemoglobin A1c (HbA1c) were collected from their medical records. Patients with uncontrolled diabetes (HbA1c > 7) were given insulin and/or oral antidiabetic drugs. Patients with other concurrent systemic illnesses were managed accordingly.

In our study, we compared these patients to age-matched COVID-19-positive controls who did not develop any fungal infection during the period of their hospital stay and for two to four weeks of their discharge and studied patients’ profiles including the presence of comorbidities and treatment received.

We addressed factors that could be associated with the development of fungal infection and studied the period between COVID-19 positivity and the onset of ROCM. Statistical analysis was done using the Chi-square test. A p-value < 0.05 was considered significant.

## Results

The total number of patients enrolled in the study was 30. The study population comprised 15 patients as cases of mucormycosis and 15 patients as COVID-19-positive controls. The age of the patients in both groups ranged from 40-60 years. There were 13 females and 17 males in the study. No statistical correlation between age and gender (p-value > 0.05) was found.

Table 1 shows the risk factors associated with mucormycosis. All the patients in the control group had clinical features suggestive of COVID-19 infection and were positive on RT-PCR. However, among patients who presented with mucormycosis, all had a history suggestive of COVID-19 infection, though only 11 (73.3%) patients had a positive RT-PCR report. The reason that four (26.7%) patients never tested positive by RT-PCR may be due to the low sensitivity of this test as it has some limitations. However, at the time of presentation of ROCM, all patients tested negative by RT-PCR.

**Table 1:** Risk factors associated with mucormycosis RT-PCR: Reverse transcription polymerase chain reaction COVID-19: Coronavirus disease

		Controls	Mucormycosis cases	Total number of patients	P-value
Reverse transcription-polymerase chain reaction (RT-PCR) result	Negative	0	4	4	0.032
	Positive	15	11	26	
Oral corticosteroids	Yes	15	15	30	NA
Intravenous corticosteroids	No	0	4	4	0.032
	Yes	15	11	26	
Injection remdesivir	No	1	5	6	0.068
	Yes	14	10	24	
Mechanical Ventilation	No	12	15	27	0.068
	Yes	3	0	3	

It was found that all our study participants had a history of intake of oral corticosteroids whereas intravenous corticosteroids were received by all controls and 11 (73.3%) of the patients with mucormycosis and found to be significantly associated (p-value = 0.032) with both, oral and intravenous corticosteroids. On investigating the correlation of remdesivir injection with the development of ROCM, we found that all controls received this drug with the exception of one patient who had severe hepatic disease, whereas among patients who developed mucormycosis five (33.3%) did not receive remdesivir and no significant correlation was found (p-value = 0.068).

Among controls, three (20%) patients were subjected to mechanical ventilation during their hospital stay as compared to patients with mucormycosis who did not receive any ventilatory support, and therefore it was also found to be statistically insignificant (p-value = 0.068).

The mean duration from COVID-19 positivity to the presentation of mucormycosis was 12.10±7.27 days, with a range between eight to 42 days. Concurrent ongoing COVID-19 infection with ROCM was not observed in our study.

Table 2 compares the co-morbidities between cases and controls. It was noted that out of the total patients, all patients with mucormycosis and four (26.6%) patients in the control group had uncontrolled blood sugar as suggested by their HbA1c values and this was found in our study as the most significant correlation (p-value = 0.003).

**Table 2:** Presence of comorbidities CAD: coronary artery disease; CKD: chronic kidney disease;  HTN: hypertension

	COVID-19 positive controls	Mucormycosis cases	Total	P-value
Diabetes	4	15	19	0.003
Anemia	1	0	1	
CAD	1	0	1	
CKD and HTN	2	0	2	
HTN	6	5	11	
Leukemia	1	0	1	
Total	15	15	30	

Co-morbidities like anemia, chronic kidney disease (CKD), coronary artery disease (CAD), and leukemia were found in controls, but none of these conditions were seen in patients developing mucormycosis.

Table 3 depicts the risk estimation with different parameters. The risk of mucormycosis was found to be 2.667 times higher in diabetics, and 1.364 times higher in patients who received intravenous corticosteroids and in those having positive RT-PCR. Use of remdesivir injection was found to have no protective effect.

**Table 3:** Risk estimation with different parameters RT-PCR: Reverse transcription polymerase chain reaction

Parameters	Odds ratio for mucormycosis	Confidence interval
Using intravenous corticosteroids	1.364	1.005-1.850
Using injection remdesivir	0.143	0.014-1.418
Diabetes mellitus	2.667	0.521- 13.655
RT-PCR positive	1.364	1.005- 1.850

Table 4 compares mean HbA1c levels with the development of mucormycosis. On the Independent samples t-test, HbA1c was significantly higher in those with mucormycosis (10.4733±2.46%) compared to HbA1c of those in the control group.

**Table 4:** Comparing mean hemoglobin A1c (HbA1c) levels with mucormycosis status

	Mean HbA1c level	Standard deviation	Standard error of the mean	P-value
Mucor cases	10.4733	2.46387	0.63617	<0.001
COVID-19 positive controls	5.9800	0.88657	0.22891	

In the following Table 5, calculating the binomial regression, the odds ratio of 13.678 shows that patients with an abnormal HbA1c level have a 13.678 times higher risk of getting infected with mucormycosis.

**Table 5:** Binomial regression HbA1c: hemoglobin A1c B: coefficient for the constant S.E.: standard error around the coefficient for the constant Wald: Wald Chi-Squared Test Df: degrees of freedom OR: Odds ratio

Variable	B	S.E.	Wald	Df	P-value	OR
Age	-0.111	0.122	0.828	1	0.363	0.895
Gender	1.924	2.504	0.590	1	0.442	6.848
HbA1c	2.616	1.188	4.850	1	0.028	13.678
Constant	-13.000	8.221	2.500	1	0.114	0.000

## Discussion

The incidence of mucormycosis increased rapidly in the COVID-19 pandemic scenario. During the second wave of the pandemic, ROCM has claimed many an eye and a life. If COVID-19 is here to stay, or the world faces more waves of the uprising of this infection, we should be better prepared to tackle secondary infections like mucormycosis in the future which may grow to epidemic proportions. Rhino-orbito-cerebral mucormycosis is a potentially lethal infection and diabetes mellitus poses a major risk factor.

While COVID-19-associated pulmonary aspergillosis has received much international attention, the Indian epidemiology of invasive mold infections in the intensive care unit reveals a significant burden of mucormycosis [9].

Our study on patients with ROCM presenting to a level 3 COVID-19 hospital during a period of two months showed the mean age of the patients to be 50.6±6.28 years with a skew deviation towards the male gender (60%) vis-à-vis female (40%).

A statistically significant association between mucormycosis and the presence of diabetes was noted in the present study. While we studied most of the systemic factors as well as the treatment modalities in COVID-19-infected patients, the most marked risk factor for the development of ROCM in our study was found to be the presence of diabetes mellitus so much so that an HbA1c value of >8% was found to be associated with 13.67 times higher risk of development of this dreaded fungal infection.

In a study by Patel et al., diabetes mellitus was found to be the most common predisposing factor (73.5%). This is in accordance with our findings [10]. They conducted a multicentric study at 12 tertiary care centers across India and reported that disseminated and rhino-orbito (with cerebral extension) mucormycosis, shorter duration of symptoms, shorter duration of antifungal therapy, and treatment with amphotericin B deoxycholate (vs. liposomal) were independent risk factors for mortality.

Results of some other studies, like one by Sen et al., also showed that the major risk factor present in their cases with ROCM was uncontrolled type 2 diabetes [11].

We found that COVID-19 positivity and concurrent steroid use further decrease immunity and increase the risk of fungal disease 1.364 times. This correlation has also been reported in a study conducted by Ravani et al. who showed that 61.2% of their mucor patients had a history of use of intravenous methylprednisolone [12]. In our study, both cases and controls had a history of use of oral steroids whereas intravenous steroid use was also present in 11 out of 15 (73.3%) mucormycosis patients and all COVID-19-positive controls (p-value = 0.032).

ROCM can cause significant morbidity in patients depending on its severity. We chose to adopt the staging system proposed by Honavar [8]. Most of our patients (46.7%) presented at stage 3c where apart from bilateral paranasal sinus (PNS) involvement there was orbital involvement with varying degrees of vision loss. One of the patients (6.67%) in our study expired due to cardiorespiratory arrest within 20 days of diagnosis of ROCM, whereas six out of 15 (40%) patients developed irreversible vision loss. Nasal endoscopy-guided debridement was done for all the cases while one needed radical orbital exenteration. In a case series reported by Sarkar et al., four patients expired within one month of diagnosis and five had irreversible vision loss [13]. Early establishment of the diagnosis and prompt surgical intervention can aid in controlling the extent and severity of the disease.

We also studied other co-morbidities present in COVID-19 patients like hypertension, chronic kidney disease, leukemias, etc., and found no significant correlation of any of these with the development of mucormycosis. However, none of the COVID-19 patients in our control group had any major immunocompromised condition like human immunodeficiency virus (HIV) infection, organ transplant, etc., which could also increase the risk, such as reported by Song et al. who showed that COVID-19 infection accompanied by immunocompromised states like HIV infection and severe prolonged neutropenia increases the likelihood of developing fungal co-infection [14].

Our study also found that a history of ventilator support does not increase the risk of development of mucormycosis (p-value = 0.068) since three out of 15 (20%) controls who were on ventilator support did not develop any signs of mucormycosis infection.

Our study showed that the mean time of onset of mucormycosis symptoms from COVID-19 diagnosis was 12.10±7.27 days, with a range between eight to 42 days. Comparable results were reported by Sen et al., where in their study mean duration between diagnosis of COVID-19 and development of symptoms of mucor was 15±9.6 (3-42) days [11].

In a case report by Mehta and Pandey, the study patients with coronavirus infection developed mucormycosis during the course of illness, but none of the cases of ROCM taken for our study were COVID-19 RT-PCR positive at the time of diagnosis. We also found a lack of development of mucormycosis in patients with concurrent COVID-19 illness taken as controls [15].

This is a retrospective study where we found an association between positive COVID-19 RT-PCR report, use of systemic steroids, and presence of uncontrolled diabetes. However, a study done on a larger scale would have been more ideal and determinative.

It is noteworthy to mention that our study is limited by the short follow-up and geographical localization of cases. Underlying conditions and risk factors might have been underreported.

## Conclusions

COVID-19-associated ROCM predominantly affects middle-aged men and women, with the majority of patients developing onset of ROCM symptoms between eight to 42 days from diagnosis of COVID-19. Aggressive medical and surgical management is critical, and patients will require co-management by multiple specialties. Collaboration between ophthalmology, otorhinolaryngology and radiology is important.

Judicious use of steroids and strict control of blood sugar levels should be emphasized in the management of COVID-19 patients. A high index of suspicion especially in diabetics and early diagnosis and management of ROCM at a lower grade of severity can help save patients from mortality and loss of eye(s). Use of appropriate and aggressive antifungals and surgical debridement is advocated. Diabetes mellitus and corticosteroids are important and independent risk factors for COVID-19-associated ROCM.

The authors recommend using prophylactic treatment protocols for the same and establishing guidelines, especially for high-risk patients to reduce morbidity and mortality.
